# "The Hospital" Medical Book Supplement.—No. XXIII

**Published:** 1909-11-20

**Authors:** 


### "THE HOSPITAL" MEDICAL BOOK SUPPLEMENT.—No. XXIII.

CONTENTS.

| | Page |
|---|---|
| **The Lymphatic System and Tuberculosis** | 211 |
| **Medicine—** | |
| The Morphia Habit and its Voluntary Renunciation | 212 |
| A System of Clinical Medicine for Students and Practitioners | 212 |
| The Open-Air or Sanatorium Treatment of Pulmonary Tuberculosis | 212 |
| Studies in Tuberculosis | 212 |
| Infectious Diseases | 212 |
| Lectures on Hysteria and Allied Vaso-motor Conditions | 213 |
| **Mental Diseases—** | |
| On the Inheritance of the Diatheses of Phthisis and Insanity | 214 |
| **Chemistry and Materia Medica—** | |
| An Introduction to Practical Chemistry | 213 |
| A Manual of Forensic Chemistry, dealing especially with Chemical Evidence | 213 |
| Materia Medica, Pharmacy, Pharmacology, and Therapeutics | 213 |
| **Miscellaneous Items—** | |
| The Life of Sir William Broadbent, Bart., F.R.S. | 214 |
| Sight Testing Made Easy | 214 |
| Air and Health | 214 |

### THE LYMPHATIC SYSTEM AND TUBERCULOSIS.

It is well-known that when the tubercle bacillus invades the human body, its line of advance, like that of other micro-organisms, is frequently along the lymphatics; the battles and skirmishes, as it were, being fought at the lymphatic glands themselves. The evidence establishing this is mainly derived from cases in which a certainly tuberculous infection, occurring in a given region, is followed by inflammation of the lymphatic glands draining that region —such lymphatic glands on excision proving to be tuberculous. Those who have read Cornet's well-known work will recall many interesting examples. Further, this author has shown that the tubercle bacillus can penetrate mucous membranes without leaving a local lesion, and that thus the first sign of infection may be glandular swelling. Now, although the study of phthisiogenesis, to use a handy term, has been much furthered by the discovery of these facts, yet it is probable that they have been too much drawn upon. Not a few writers have over-estimated the range of access possible by the lymphatic route. To any such offenders a perusal of Dr. Most's study* should be beneficial.

The writer begins with the just remark that until lately exact topographical investigations with modern technique, having for their object delimitation of the regional lymphatic areas of most interest in connection with tuberculosis, did not exist; and he recalls some elementary points respecting the lymphatic smaller vessels and capillaries which are too often forgotten by the pathologist. Just as the veins, so, too, the lymphatics have valves which determine the direction of lymph flow. It is impossible, except by violence, to inject lymphatics the reverse way of the flow—" up stream," as it were—and therefore it is quite unlikely that tuberculous infection can spread in that direction, save in the presence of grave and rapidly fatal disease or of widespread lymph stasis. These anatomical points, together with a consideration of certain facts established by general clinical experience (*e.g.* with regard to the spread of malignant disease), make the author postulate that the common occurrence of spread of infection from one lymphatic area to another must be regarded as impossible in the absence of demonstrable channels of infection, or of clear signs of extension *per continuitatem*. If made, this is an important admission, because in Dr. Most's experiments careful injection, made, with every precaution against error, on the bodies of new-born children and of adults, and on anæsthetised animals, failed to show connecting links between the bronchial and mediastinal glands on the one side, and on the other either the deep cervical group—so often affected in scrofula—or the mesenteric and retro-peritoneal, which drain the intestine. A purely lymphogenous origin of consumption, says Dr. Most, is inconceivable. The only path by which tubercle bacilli from either of the above localities can reach the lung is by entering the venous system, and travelling by way of the right heart and lesser circulation: this path he considers unlikely because thereby the invading micro-organisms have to run the gauntlet of a considerable array of the host's natural powers of resistance. The objection, however, though it may be valid enough, is clearly an *a priori* one; and it should be remembered that in animal experiments carbon particles have been described as getting to the lung with some speed by this very route. Still, as Dr. Most shows, the lymphatic system, in dogs and guinea-pigs, at any rate, is much more permeable than it is in man. Since then the purely lymphatic way is anatomically impossible, and the lymphohæmic one beset with such dangers to the bacillus, it follows that in pulmonary tuberculosis infection is commonly by inhalation. The conclusion is rather large in proportion to the body of evidence, and one turns to the author's statements respecting scrofula. It is the anterior sub-group of the deep cervical glands, he says, which is most commonly affected, and this division drains directly the posterior and lateral pharyngeal walls, and therefore also the faucial tonsil, a structure which, by its site, and the anatomical conformation of its epithelial covering, is especially likely to become infected with tubercle. The absence of obvious local affection is well explained by the observations of Cornet, already mentioned. In scrofula, then, it is most often the tonsil which is at fault. But from scrofula to consumption, or from abdominal tuberculosis to consumption, the path of infection is a very round about one.

It must be remembered that a great deal of recent experimental work by Whitla and others points to conclusions exactly the opposite of those of this author; so that the matter must still be regarded as an open question.

* A. Most, *Die Topographie des Lymphgefässaparates des menschlichen Körpers und ihre Beziehungen zu den Infektionswegen der Tuberkulose.* Bibliotheca Medica, *Abteilung C, Hft.* 21.

### MEDICINE.

The Morphia Habit and its Voluntary Renunciation. By Oscar Jennings, M.D. (Paris). (London: Messrs. Baillière, Tindall, and Cox. 1909. Pp. x.-492. Price 7s. 6d.)

This volume consists of a personal relation of the suppression of the morphia habit after the patient had been addicted to it for twenty-five years. We have not any statistics to hand, but we certainly demur to the author's statement "that one medical man out of four is a drug habitué, usually a morphinist, that the proportion of medical addicts to the total of cases is in some statistics as high as 90 per cent., and that one-fifth of the mortality in the profession is said to be caused by morphinism"; and we feel that even strong points lose a good deal of their force when they are in any way overdrawn. Nevertheless, even though the morphia habit were very much rarer than the author states it to be, the personal account of a patient who is a medical man, and who succeeded in curing himself of the complaint after it had been well established for so many years, must necessarily command the interest and the respect of the profession. The author holds that every patient suffering from morphinism is really curable, if only his own abortive attempts at emancipation are properly seconded by efficient encouragement and treatment. This treatment needs to be extremely minute in its directions; and the work before us contains as detailed an account of the methods that are likely to prove successful as any we know of.

A System of Clinical Medicine for Students and Practitioners. By Thomas Dixon Savill, M.D. (London: Edward Arnold, 1909. Pp. xxviii-963, with four coloured plates and 172 illustrations. Price 25s. net.)

This is a second edition of an already well-known work which first appeared in 1903. The revision has been made by Dr. Savill himself, assisted by Dr. Frederick S. Longmead and Dr. Agnes F. Savill. We think it a great improvement to have the work in one volume, as at present, instead of in two as in the first edition.

One of the main features of the book is that it deals essentially with clinical medicine; that is to say, the diagnosis of different symptoms and prognosis in different cases, and the treatment that may be adopted under different circumstances, are dealt with fully, but pathology is touched either not at all or in the briefest manner, and only where it helps more clearly the diagnosis, prognosis, or treatment. The illustrations are good. There are many points to which we might take exception in the book; for instance, we do not see why so important a disease as pernicious anæmia should be relegated to the small print, in which the rarest of diseases, such as bronzed diabetes, are described, nor why it is not allowed a little more than a single page. Similarly, in Addison's disease and many other complaints that are well known in general practice one would have hoped for fuller information, even though this necessitated omissions from other parts of the book. Notwithstanding this, and in spite of the large number of medical textbooks that are at present available, we feel confident in recommending this one.

The Open-air or Sanatorium Treatment of Pulmonary Tuberculosis. By F. Rufenacht Walters, M.D., F.R.C.S., Physician to the Crooksbury Sanatorium. (London: Baillière, Tindall and Cox, 1909. Pp. xvi-323. Price 5s. net.)

This book is divided into two parts: the first comparatively non-technical, so as to be understood by the laity as well as by the profession; the second technical, and intended for the profession rather than the laity. The author has succeeded well in putting into book-form a very large number of simple yet practical and very important points, without attention to which patients suffering from pulmonary tuberculosis are not being placed on the very best basis for the bringing about of their effectual cure. As is pointed out here, the sanatorium treatment of consumption does not consist solely in the administration of fresh air. There are the questions of clothing, of baths and ablutions, of recreation, of travel, what to eat and when to eat it, the necessary variations there must be made in the diet according to circumstances, and scores of other similar points upon which it is not always easy to advise the patient unless he himself has some fairly clear idea of the object that each regulation suggested by the doctor has in view.

In the technical part of the volume a large number of practical suggestions are made not only in regard to quite modern methods of treatment by tuberculin, opsonic index determinations, and so forth, but also by older methods—climatic, hygienic, dietetic, and medicinal. We do not agree with the author on every point; for instance, we hold that there are many cases, especially of early phthisis, in which it is better to send the patient anywhere in the country or to the seaside rather than to a sanatorium. We do not hold that it is essential for rectal temperatures to be taken in every case; and there are other points in which we differ from the writer; nevertheless we find his book most lucid, practical, and to be recommended.

Studies in Tuberculosis. By Henry Clarke, M.A., M.D. (London: Archibald Constable and Co., Ltd. 1909. Pp. 59. Price 5s. net.)

This publication consists in the main of a thesis for the degree of Doctor of Medicine of Cambridge University. It does not, we think, contain any practical matter which is not obtainable in other works that cost little, if any, more, and yet contain much more information than does Dr. Clarke's thesis. We are interested to note that he regards the temperature as a sufficient guide in the use of tuberculin without the necessity of opsonic index determinations; in this respect the author agrees with what has already been pointed out by Dr. Latham and others. We think that upon the whole the opinions given err on the side of being too drastic, as exemplified, for instance, by the following paragraph:—

"So far as prevention of consumption is concerned, reliance must be placed on the suppression of tuberculosis in cattle, and in the segregation of consumptive patients who by their habits are dangerous to their neighbours; this can be done efficiently only by public bodies, although compulsion will probably not be necessary, except in a very small class of patient."

Infectious Diseases. By Claude Buchanan Ker, M.D., F.R.C.P. (London: Henry Frowde, Hodder and Stoughton. 1909. Pp. 555, with 24 plates. Price 20s. net.)

This is another of the Oxford medical publications, and it maintains their general high standard in the matter of printing, paper, and illustrations. The subjects dealt with are: measles, rubella, scarlet fever, smallpox, vaccinia, chicken-pox, typhus fever, relapsing fever, enteric fever, diphtheria, erysipelas, whooping-cough, mumps, and cerebro-spinal meningitis. The illustrations have been taken from actual cases, and they have the advantage of being taken from fairly common types rather than from those which have attracted particular attention owing to their rarity. The Edinburgh City Hospital has the great advantage from a teaching point of view that it admits not only the notifiable fevers, but also measles, rubella, mumps, and whooping cough, so that the chapters upon these are particularly noteworthy, seeing that they are written by one of the few persons who have had a large hospital experience of these common maladies. The author deals with his subject mainly from the point of view of diagnosis, prognosis, and treatment, saying relatively little as to pathology and bacteriology, except where these have an obvious practical bearing. Practitioners will find the work an admirable one, especially in the matter of treatment and dietetics; for medical students the work is too large, but for those qualified and in practice it is to be highly recommended.

Lectures on Hysteria and Allied Vaso-motor Conditions. By Thomas Dixon Savill, M.D. (Glaisher: 1909. Pp. 262.)

As will be noted from the title of the book, hysteria is claimed as essentially a vaso-motor condition. Present-day physicians will be curious to know how such a retrograde conclusion is reached. The explanation appears to be that the theory is built round a diagram, which represents the central idea in the author's mind. He regards a hysterical attack as a "splanchnic storm" in which the abdominal viscera become hyperæmic, and the brain correspondingly anæmic. Why are the abdominal organs hyperæmic? No observations are recorded on the blood-pressure, but—here comes in the diagram. To quote the author (p. 29): "The solar plexus is connected with the greater and lesser splanchnic nerves and communicates with the spinal roots *including the first lumbar nerve, from which the ilio-hypogastric and ilio-inguinal nerves spring.*" (Italics are the author's.) . . . "The reflex vaso-motor centres in the solar plexus may, therefore, be affected by afferent stimuli (a) from the brain, (b) from the inguinal region along the ilio-hypogastric or ilio-inguinal nerves, and (c) any of the other afferent nerves." Why he happens to select the first lumbar nerves as of special importance in connection with the solar plexus Dr. Savill does not explain, though he makes a great point of that particular root supplying the area in the inguinal region, which is not infrequently capable of inducing a hysterical fit. That pressure on this spot may also arrest a hysterical fit does not affect his chain of reasoning, nor the fact that ordinary syncope is unassociated with hysterical phenomena. Further (page 34) he states that "the ilio-hypogastric nerve is apparently the centripetal depressor nerve of the abdominal sympathetic." The author proceeds to discuss the pathology of hysterical attacks, and would have one believe that the complex psychomotor phenomena of a hysterical attack are the result of cerebral anæmia or hyperæmia (which of the two he is not always clear), according as the splanchnic area is hyperæmic or the reverse.

Later on, an attempt is made to explain hysterical anæsthesiæ and contractures, of obvious psychogenic origin, as due to vaso-motor affection of particular areas of the brain. Thus on page 101 a typical segmental anæsthesia is ascribed to a vaso-motor affection of the "sensory crossway" in the internal capsule! The author talks of "hysterical tics," apparently unaware that the recent work of the French school has established the thesis that tics are no part of hysteria, but form one of the stigmata of psychasthenia. Examples like these might be multiplied, but from what we have pointed out, it will be seen that this book gives no up-to-date insight into the modern problems of hysteria.

### CHEMISTRY AND MATERIA MEDICA.

An Introduction to Practical Chemistry. By A. M. Kellas, B.Sc., Ph.D. (London: Henry Frowde, Hodder and Stoughton. 1909. Pp. 262. Price 3s. 6d. net.)

This book is designed by its author, who is lecturer on chemistry at the Middlesex Hospital Medical School, especially to meet the requirements of the examination by the conjoint boards of the Royal Colleges of Physicians and Surgeons in London. We are loath to say that the volume before us excels older and smaller books upon the subject which have been long familiar; but it is an eminently useful work, and one which is likely to be of practical service in assisting students to learn their chemistry for examination purposes. There is one considerable objection to it, however, namely that it is much too large to be carried in the pocket; laboratory handbooks should, whenever possible, be of small size.

A Manual of Forensic Chemistry, dealing especially with Chemical Evidence. By William Jago, F.I.C., F.C.S., Barrister-at-Law. (London: Stevens and Haynes, Law Publishers, Bell Yard, Temple Bar.)

We have read this book with pleasure, for it seems to us that it supplies a long-felt want very admirably. Mr. Jago, who is well known as an authority on chemistry, has compiled the work from notes of a course of special lectures on Forensic Medicine delivered recently at University College, and he is to be congratulated upon the lucidity and terseness with which he has managed to convey essentials to the reader. Every medical student should read the book, and we venture to suggest that it is a work that should find a place on the shelves of every medical practitioner. It deals very fully with such important matters as food adulteration, criminal and civil actions, preservatives, and colouring matters, and, what is specially important, it gives very valuable hints as to legal procedure, the law of evidence, and such like matters about which the average medical man knows lamentably little. A feature in it is the citation of leading cases and authorities, and the clear indications for lines of attack and defence in various cases. There are a few obvious errors, and some expressions of opinion with which experts may not entirely see eye to eye with the author; but as a whole the book is beyond censure, and we heartily commend it to general practitioners, who will find it a veritable mine of information so far as medical evidence in adulteration cases is concerned. To medical officers of health it should prove indispensable as a handy reference book.

Materia Medica, Pharmacy, Pharmacology, and Therapeutics. By W. Hale White, M.D., F.R.C.P. (London: J. and A. Churchill. Eleventh Edition. 1909. Price 6s. 6d. net.)

If there is any one book on which the opinion of medical students is unanimous, surely it must be the familiar volume in an olive-green binding, known colloquially as "Hale White." Of its popularity, the issue of eleven editions in seventeen years is evidence enough; though it is curious to note that, whereas five years were required to exhaust the first edition, ten more have been demanded in the twelve years since the appearance of the second edition. And this popularity has been won by really sound work, not by any striving after the methods of the examination cram-book. In this new edition a good deal of fresh material is incorporated, but the general plan remains the same as before. In points of detail there are here and there additions or omissions which individual experience might desire to see adopted. In the discussion of leeches, for instance, no mention is made of their almost specific action in dispelling the acute pleuritic pain of lobar pneumonia. The extensive use of guaiacol carbonate for chronic articular lesions might well receive more notice than the two lines in which the subject is referred to. Urinary antiseptics also are dismissed in somewhat cursory fashion, both in the preliminary section on general pharmacology and therapeutics, and also in the more detailed later section on organic materia medica. But these are minor blemishes on a book which is and has long been a standard work. It were greatly to be wished that medical practitioners had time to read through this new edition; for, if the experience of the present reviewer is any guide, they would be reminded of many half-forgotten wrinkles in pharmacology and therapeutics which are well worth knowing and turning to practical advantage.

### MENTAL DISEASES.

On the Inheritance of the Diatheses of Phthisis and Insanity. A Statistical Study, Based upon the Family History of 1,500 Criminals. By Charles Goring, M.D., B.Sc. Drapers' Company Research Memoirs. (London: Dulau and Co. 1909. Pp. 28. Price 3s.)

The criminal class was deliberately chosen as the subject of these investigations, because the records of the inmates of hospitals and asylums, in whose families at least one member is diseased, cannot be taken fairly as representative of the general population. The results lead the author to the following conclusions:—(1) The tuberculous diathesis is inherited at the same rate as all other physical characters in man. (2) The prevalence of tuberculosis in the population lies between eight and ten per cent., and is probably nearer the lower figure. (3) There is no evidence of marital infection in the class dealt with. (4) There is no evidence that the correlation between parents and offspring is greater in the poorer classes, where environment would increase the liability to infection. This is consistent with the correlation being largely hereditary in character. (5) The importance of the hereditary factor, as opposed to direct contagion, is shown by the fact that the prevalence of phthisis in children of infected fathers and mothers is equal, and that its prevalence among workers in consumptive hospitals is not greater than among individuals of the general population with the same degree of diathesis. (6) Criminal data show that the inheritance of the insane diathesis presents a correlation between parents and offspring sensibly the same as that in phthisis—a conclusion which renders the contagious theory of the origin of phthisis difficult to uphold. (7) The prevalence of insanity is greater than the value assumed by Heron (Eugenics Laboratory Memoirs, III. Dulau and Co.), or else is greater in the criminal stock as distinguished from the normal population. The memoir bears the impress of careful and painstaking labour, and is worthy of perusal by those interested in the study of eugenics.

### MISCELLANEOUS.

The Life of Sir William Broadbent, Bart., F.R.S. Edited by his daughter M. E. Broadbent. With portrait. (London: John Murray, Albemarle Street, W. 10s. 6d. net.)

There are all too few biographical memoirs of prominent medical men, and this work, brief as it is and in many respects wholly unsatisfying, is to be welcomed as an interesting contribution to their number. As a leader of the profession in England, who enjoyed an almost international reputation, Broadbent deserved a fuller biography than this somewhat scrappy compilation of extracts from letters strung together with a very thin string of explanatory notes. As it is, we must be content with the memoir. There were reasons, doubtless, that prevented a fuller and more generally interesting epitome of his life and work from being published, and his many friends and admirers will be grateful for even this short tribute to his memory. So far as it goes, the Life is excellent. It omits nothing relevant, and it wastes no space on descriptions of the obvious. Good judgment has been shown by the editress in selecting the most usefully interesting excerpts from the letters, and the excess of purely private matter, especially the religious references, may be pardoned when it is remembered that the memoir was in the first instance published for private circulation only. Moreover, they show a side of the late Sir William's character which is interesting and instructive. Some of the descriptions—for instance, in the letters from the Franco-German battlefields—are worthy of a good writer, if not a great one, and the whole story of Broadbent's life, as here told, should appeal to every member of the profession. His early struggles were not more severe than many another public man's, and success came sooner to him than to most. Nevertheless the recital of those early days is well worth reading, and it is interesting to note that his first candidature for appointments involved his adhesion to that absurd and immoral system which still prevails at some hospitals and dispensaries—canvassing. The book is a very short one, easily read in an evening, and will well repay reading.

Sight Testing Made Easy. By W. W. Hardwicke, M.D. (London: J. and A. Churchill. 1909. Pp. 66. Price 2s. 6d. net.)

This modest brochure contains no account of methods of sight testing requiring the use of the ophthalmoscope, which seems a defect. For any who can grasp the instructions in sight testing under cycloplegics as herein laid down, and are to be trusted with the use of such drugs, might just as well learn straight away the ordinary methods of refraction for the estimation of visual defect. Apart from this there is little fault to be found with Dr. Hardwicke's monograph, which is practical as far as it goes. In one or two passages the author says what we are quite convinced he does not mean to say; and we are also sceptical of the value of counter-irritation applied to the temples and blisters or solution of iodine behind the ears as remedies for progressive myopia.

Air and Health. By Ronald C. Macfie. (Methuen and Company. 7s. 6d. net.)

This book deals with the physical and chemical properties of air in relation to health and disease. Ventilation is considered both from a private and public standpoint. There is a chapter on the physiology of respiration, and others treat of dust, fog, germs, etc. To the general practitioner the book will prove of but little interest or use, but to anyone who is devoting time and energy to the hygienic questions of the day, it may be of service.

